# Renal Allograft Torsion: US and CT Imaging Findings of a Rare Posttransplant Complication

**DOI:** 10.1155/2016/4273780

**Published:** 2016-03-20

**Authors:** Rohit Dewan, Anil K. Dasyam, Henke Tan, Alessandro Furlan

**Affiliations:** University of Pittsburgh Medical Center, 200 Lothrop Street, Presby East Wing, Suite E174, Pittsburgh, PA 15213, USA

## Abstract

Vascular torsion is a rare renal transplant complication which requires prompt diagnosis and surgery to salvage allograft function. We report here a case of renal allograft torsion with interesting imaging findings on unenhanced CT and color Doppler ultrasound. A 60-year-old woman with a history of pancreas and kidney transplant presented to the emergency room with nausea, vomiting, abdominal pain, and minimal urine output. Unenhanced CT of the abdomen demonstrated an enlarged and malrotated renal allograft with moderate hydronephrosis. Color Doppler ultrasound demonstrated lack of vascularity within the allograft. The patient was taken urgently to the operating room where the renal allograft was found twisted 360 degrees around the vascular pedicle. After the allograft was detorsed, the color of the kidney returned and the Doppler signals for arterial flow improved. Intraoperative biopsy showed no evidence of infarct or acute cellular rejection. The detorsed kidney was surgically fixed in position in its upper and lower poles. Follow-up ultrasound 1 day later demonstrated normal blood flow to the renal allograft and the serum level of creatinine returned to normal.

## 1. Introduction

Torsion is a rare complication of renal transplants and can be seen with both intraperitoneal and extraperitoneal allografts [1]. Clinical presentation and diagnostic imaging are nonspecific and often result in delayed surgical exploration [2]. When posttransplant deterioration in renal function occurs in the immediate postoperative setting or years later, renal torsion should be considered in the differential diagnosis. We present a case of renal allograft torsion with imaging findings on CT and color Doppler ultrasound.

## 2. Case Report

**Figure 1:**
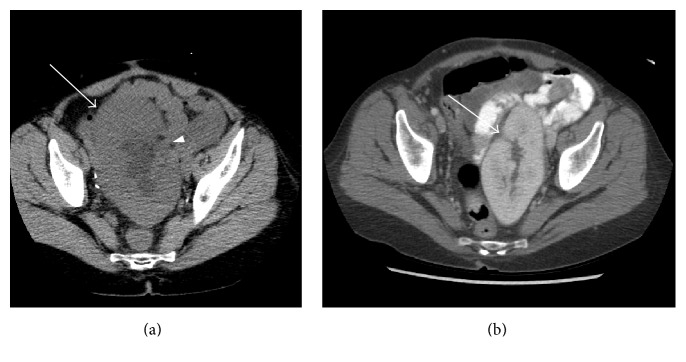
Noncontrast axial CT image through the pelvis (a) showed an enlarged left lower quadrant renal allograft (arrow) measuring 14.4 × 7.6 cm with moderate hydronephrosis and trace pelvic free fluid. Note that the hilum is directed laterally (arrowhead). This was a clear change in orientation when compared to a contrast enhanced CT scan obtained a year and half earlier (b) where the hilum was directed anteromedially (arrow).

A 60-year-old woman with history of simultaneous pancreas and kidney transplant for diabetic nephropathy presented to the ER 2 years after the transplantation for evaluation of nausea, vomiting, abdominal pain, and minimal urine output. She had an interval weight gain with BMI of 31.8 on presentation, which increased from BMI of 26.6 four months earlier. She was found to be in acute renal failure with severe increase in serum creatinine level to 9.9 mg/dL. She was hemodynamically stable and afebrile. Noncontrast CT of abdomen and pelvis demonstrated an enlarged renal allograft with moderate hydronephrosis and trace pelvic free fluid (Figure 1(a)). In retrospect, a change in orientation of the allograft was noted with the hilum directed laterally when compared to a contrast enhanced CT performed prior to torsion (Figure 1(b)) when the hilum was directed anteromedially.

**Figure 2:**
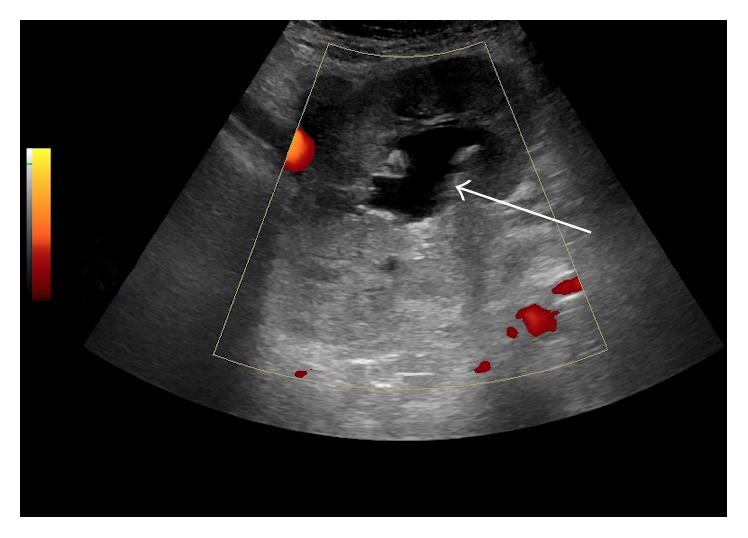
Power Doppler ultrasound demonstrates no internal vascularity within the enlarged hyperechoic left lower quadrant renal allograft raising concern for renal artery thrombosis and impending infarct. Note the moderate dilatation of the allograft collecting system (arrow).

Further evaluation with ultrasound showed an enlarged, hyperechoic renal allograft with moderate collecting system dilatation. At color Doppler evaluation only a short segment of the proximal renal artery was seen, with the distal renal artery not visualized. No blood flow was demonstrated within the allograft on color Doppler, power Doppler, and spectral analysis (Figure 2). The renal vein was not visualized. The constellation of findings was concerning for renal artery thrombosis and impending infarct.

Transplant medicine immediately took the patient to the operating room for a nephrectomy as there was concern the kidney could become necrotic and a nidus for infection. The renal allograft was found twisted 360 degrees on its vascular pedicle and ureter despite prior nephropexy, likely secondary to adhesions in its upper pole. The kidney appeared pinkish-blue indicating minimal blood flow. After taking down the band of adhesions, the allograft was detorsed and the ureter was returned to its appropriate position. A pinkish hue returned to the kidney. Intraoperative Doppler demonstrated excellent flow in the renal artery and vein. Intraoperative biopsy confirmed the kidney was still viable without evidence of infarct or acute cellular rejection. The detorsed kidney was fixed in two positions in the upper and the lower poles.

**Figure 3:**
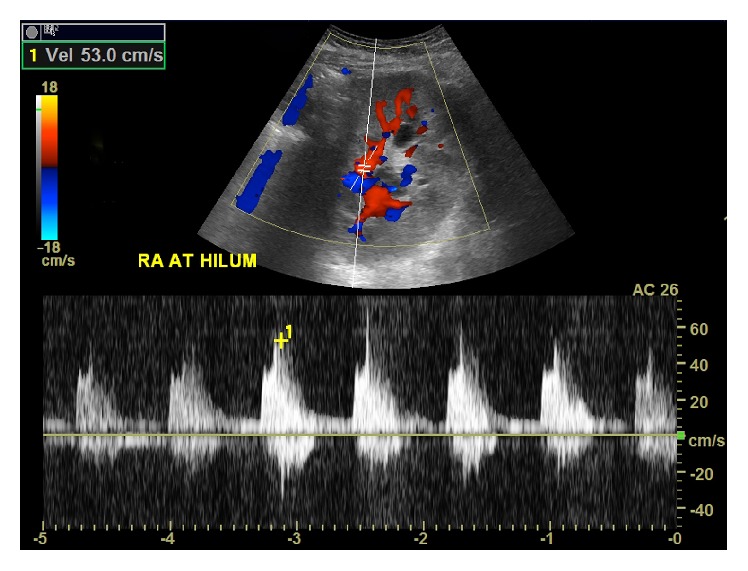
Doppler ultrasound performed on post-op day 1 after surgical detorsion demonstrated normal arterial blood flow with residual mild hydronephrosis and persistent enlargement of the renal allograft.

After surgical detorsion, all laboratory values rapidly returned to normal levels. Doppler ultrasound on post-op day 1 demonstrated normal hilar and segmental arterial and venous blood flow to the renal allograft (Figure 3).

## 3. Discussion

Renal allograft loss can occur secondary to a wide variety of etiologies including vascular, infection, rejection, and medication toxicity. Vascular etiologies can be further characterized as renal artery or vein thrombosis, renal artery aneurysm, and allograft torsion. The incidence of vascular complications ranges from 0.5% to 3.5%, with allograft torsion being the smallest subset with only 24 cases reported in the literature [2]. Torsion is defined as rotation of the allograft around the renal vascular pedicle. This results in vascular compromise which can eventually lead to parenchymal infarction. There have been fewer reported cases of extraperitoneal allograft torsion compared to intraperitoneal placement, likely due to a protective mechanism from decreased mobility in the extraperitoneal space. Of the reported cases of renal allograft torsion, 15 out of 24 have occurred with intraperitoneal placement of simultaneous pancreas and kidney transplants [2]. While torsion is much less common than other vascular complications, it should be included in the differential and diagnosed rapidly as immediate surgical intervention can salvage allograft function. Delays in diagnosis result in an increased risk of frank necrosis, acute cellular rejection, and possible transplant nephrectomy [1].

Patients with renal allograft torsion usually present with nonspecific symptoms such as nausea, vomiting, abdominal pain, decreased urine output, and elevated creatinine. Imaging thus plays a key role in the evaluation of these patients. The most suggestive finding is a change in axis of the allograft when compared to prior studies, best appreciated on cross-sectional imaging such as CT and MRI [2]. Our patient presented with a left lower quadrant allograft in the iliac fossa in which we would expect the hilum to be directed medially. Evaluating for a change in orientation on imaging is limited in cases where the allograft is twisted 180 degrees around its vascular pedicle with no change in longitudinal alignment [3].

Incomplete torsion initially results in venous compromise which can be seen on Doppler ultrasound as an elevated resistive index and elevated velocity at the main renal artery anastomosis [4]. Progression to reversed diastolic flow may occur. These findings are not specific, however, as they can also be seen with acute tubular necrosis, acute rejection, and urinary obstruction [2, 4]. As the torsion progresses, arterial compromise can result in a decreased resistive index and complete lack of blood flow on color Doppler as in our case. With ureteral compromise, the renal allograft becomes hydronephrotic, enlarged, and edematous. Vascular kinking may be easier to appreciate on contrast enhanced exams. Delayed to absent enhancement of the allograft in the corticomedullary and nephrographic phases of contrast may suggest ischemia or impending necrosis. Since patients with renal allograft dysfunction commonly present with acute kidney injury and an elevated creatinine, obtaining cross-sectional imaging with iodinated or gadolinium contrast may not be possible [2]. Ferumoxytol, an iron-based contrast agent, has been recently proposed in this population of patients for MR angiographic evaluation [2].

Treatment of renal allograft torsion requires immediate detorsion in the operating room to prevent allograft loss. Obtaining cross-sectional imaging should not delay surgical treatment when there is high suspicion for renal allograft torsion.

As torsion can be intermittent and incomplete, a normal appearing exam does not necessarily exclude the diagnosis [5]. On a study of 16 cases of intraperitoneal renal allograft torsion, 44% of allografts were salvaged after detorsion, 38% of cases resulted in nephrectomy, and 19% of cases resulted in delayed allograft loss after detorsion [6]. Data is limited regarding salvage rates of extraperitoneal torsion given that only 3 cases have been reported in the literature. Of these cases, however, all resulted in salvage of the allograft without the need for nephrectomy [1].

We report a rare case of intraperitoneal renal allograft torsion. This is a rare but known complication of renal transplantation that can occur in the immediate postoperative setting as well as years after surgery. When posttransplant deterioration in renal function occurs in the postoperative setting or years later, renal torsion should be considered in the differential diagnosis.

## References

[1] Sosin M., Lumeh W., Cooper M. (2014). Torsion of the retroperitoneal kidney: uncommon or underreported?. *Case Reports in Transplantation*.

[2] Sofue K., Vikraman D. S., Jaffe T. A., Chaubal G. N., Bashir M. R. (2015). Graft kidney torsion after simultaneous kidney-pancreas transplant: report of 2 cases and literature review. *Journal of Computer Assisted Tomography*.

[3] Wong-You-Cheong J. J., Grumbach K., Krebs T. L. (1998). Torsion of intraperitoneal renal transplants: imaging appearances. *American Journal of Roentgenology*.

[4] Ozmen M. M., Bilgic I., Ziraman I., Koc M. (2013). Torsion of extraperitoneally transplanted kidney: an unusual complication. *Experimental and Clinical Transplantation*.

[5] Sebastià C., Quiroga S., Boyé R., Cantarell C., Fernandez-Planas M., Alvarez A. (2001). Helical CT in renal transplantation: normal findings and early and late complications. *Radiographics*.

[6] Lucewicz A., Isaacs A., Allen R. D. M., Lam V. W. T., Angelides S., Pleass H. C. C. (2012). Torsion of intraperitoneal kidney transplant. *ANZ Journal of Surgery*.

